# Using deep learning to identify the recurrent laryngeal nerve during thyroidectomy

**DOI:** 10.1038/s41598-021-93202-y

**Published:** 2021-07-12

**Authors:** Julia Gong, F. Christopher Holsinger, Julia E. Noel, Sohei Mitani, Jeff Jopling, Nikita Bedi, Yoon Woo Koh, Lisa A. Orloff, Claudio R. Cernea, Serena Yeung

**Affiliations:** 1 Department of Computer Science, Stanford University, Stanford, CA USA; 2 Division of Head and Neck Surgery, Department of Otolaryngology, Stanford University, 875 Blake Wilbur Drive, Stanford, CA 94305 USA; 3 Department of Otolaryngology-Head and Neck Surgery, Ehime University Graduate School of Medicine, Shitsukawa, Toon, Ehime Japan; 4 Department of Surgery, Stanford University, Stanford, CA USA; 5 Department of Head and Neck Surgery, Yonsei University School of Medicine, Seoul, Korea; 6 Department of Surgery, University of São Paulo Medical School, São Paulo, Brazil; 7 Department of Biomedical Data Science, Stanford University, 350 Jane Stanford Way, Stanford, CA 94305 USA; 8 Clinical Excellence Research Center, Stanford University School of Medicine, Stanford, CA USA

**Keywords:** Thyroid gland, Thyroid diseases, Medical research, Surgical oncology

## Abstract

Surgeons must visually distinguish soft-tissues, such as nerves, from surrounding anatomy to prevent complications and optimize patient outcomes. An accurate nerve segmentation and analysis tool could provide useful insight for surgical decision-making. Here, we present an end-to-end, automatic deep learning computer vision algorithm to segment and measure nerves. Unlike traditional medical imaging, our unconstrained setup with accessible handheld digital cameras, along with the unstructured open surgery scene, makes this task uniquely challenging. We investigate one common procedure, thyroidectomy, during which surgeons must avoid damaging the recurrent laryngeal nerve (RLN), which is responsible for human speech. We evaluate our segmentation algorithm on a diverse dataset across varied and challenging settings of operating room image capture, and show strong segmentation performance in the optimal image capture condition. This work lays the foundation for future research in real-time tissue discrimination and integration of accessible, intelligent tools into open surgery to provide actionable insights.

## Introduction

Artificial intelligence (AI) is transforming clinical medicine in radiology^1,2^, pathology^3^, dermatology^4^, and ophthalmology^5^. Computer vision, the field of AI focused on automatic interpretation of visual data, has improved detection of abnormalities in chest roentgenograms^1^, pneumonia in CT scans^2^, and clinically occult nodal metastasis in breast cancer in whole-slide images^3^. In these medical specialties, standardized imaging methods and often large datasets of annotated images are already an essential element of the clinical workflow.

However, the implementation of deep learning lags behind in surgery. Despite the critical role played by visual discrimination during surgery, standardized imaging methods are currently not often integrated into surgical workflows, especially for open surgery. Still, computer vision offers a unique opportunity to assist and augment surgeons intraoperatively. For instance, thyroid surgery is one of the most commonly performed surgical procedures, with an estimated 150,000 operations per year in the United States alone^6^. Excellence and reliability in discriminating parathyroid glands from fat and distinguishing the recurrent laryngeal nerve (RLN) from small-caliber blood vessels is crucial. For expert surgeons, such visual discrimination is refined over years of practice. However, population-based data^7,8^ has found higher rates of postoperative complications when thyroidectomy is performed by surgeons who perform this procedure less frequently. In our work, we ask the questions: Could computer vision tools be developed to assist surgeons during thyroidectomy, and in particular, to identify the RLN? How do operating room image capture conditions affect such a method’s performance?

Early work investigating computer vision’s potential in surgical procedures include anatomical identification in endoscopic images; these have analyzed relatively homogeneous scenes and are primarily diagnostic in nature^9^. For instance, computer vision has been explored for disease, artifact, and anatomy detection in gastrointestinal^10–15^ and head and neck^16–21^ endoscopy. Further work has also introduced computer vision methods for use in laparoscopic and robot-assisted surgery, where surgical scenes are more complex and involve surgical activity and instruments constrained within a small field of view. These works span a wide range of tasks, including activity recognition^22–24^ and phase detection^25–28^ in surgical videos, surgical instrument detection^29–32^ and segmentation^33–35^, as well as anatomical identification in a variety of procedures^36,37^ such as laparoscopic cholecystectomy^38–40^. These works demonstrate the promise of using computer vision for surgical video analysis and intraoperative object and anatomy identification.

However, relatively few works have analyzed open surgery images, where there exists even more complexity and variability in the surgical scene than seen in laparoscopy. For example, in this setting, computer vision has only been studied in heart vessel segmentation in open-heart surgery^41^ and wound segmentation in videos of open-neck procedures^42^. The role of computer vision in open surgery remains less-studied than that in endoscopy, laparoscopy, and robotic surgery due to the higher difficulty and often lower quality of image capture during open procedures. Consequently, little-to-no work has investigated using deep learning to identify the RLN; only one recent work has investigated using augmented reality (AR) intraoperatively to register pre-computed AR images of the RLN from CT scans onto the surgical anatomy^43^.

In this study, we utilized computer vision techniques powered by deep learning to discriminate and identify critical soft tissue anatomy during thyroidectomy. We present an end-to-end algorithmic solution for nerve segmentation and measurement, and we both quantitatively and qualitatively analyze its performance. Due to the greater variety of open surgery scenes in contrast to the more controlled environments of endoscopy, laparoscopy, and robot-assisted surgery, it is also critical to understand how to manage this variation in open surgery to best enable intraoperative anatomical analysis across the diverse and challenging settings of operating room image capture. To this end, we analyze how image capture conditions impact the algorithm’s performance, with the hope that these insights will guide future work involving image capture for open surgery. In comparison to prior work, the soft tissue segmentation task in this study uses free-form, digital images and therefore faces several unique challenges, which we discuss further. This work lays the foundation for future translational study bringing AI to vision and discrimination for open surgery.

## Results

### Segmentation and measurement pipeline and evaluation

**Figure 1:**
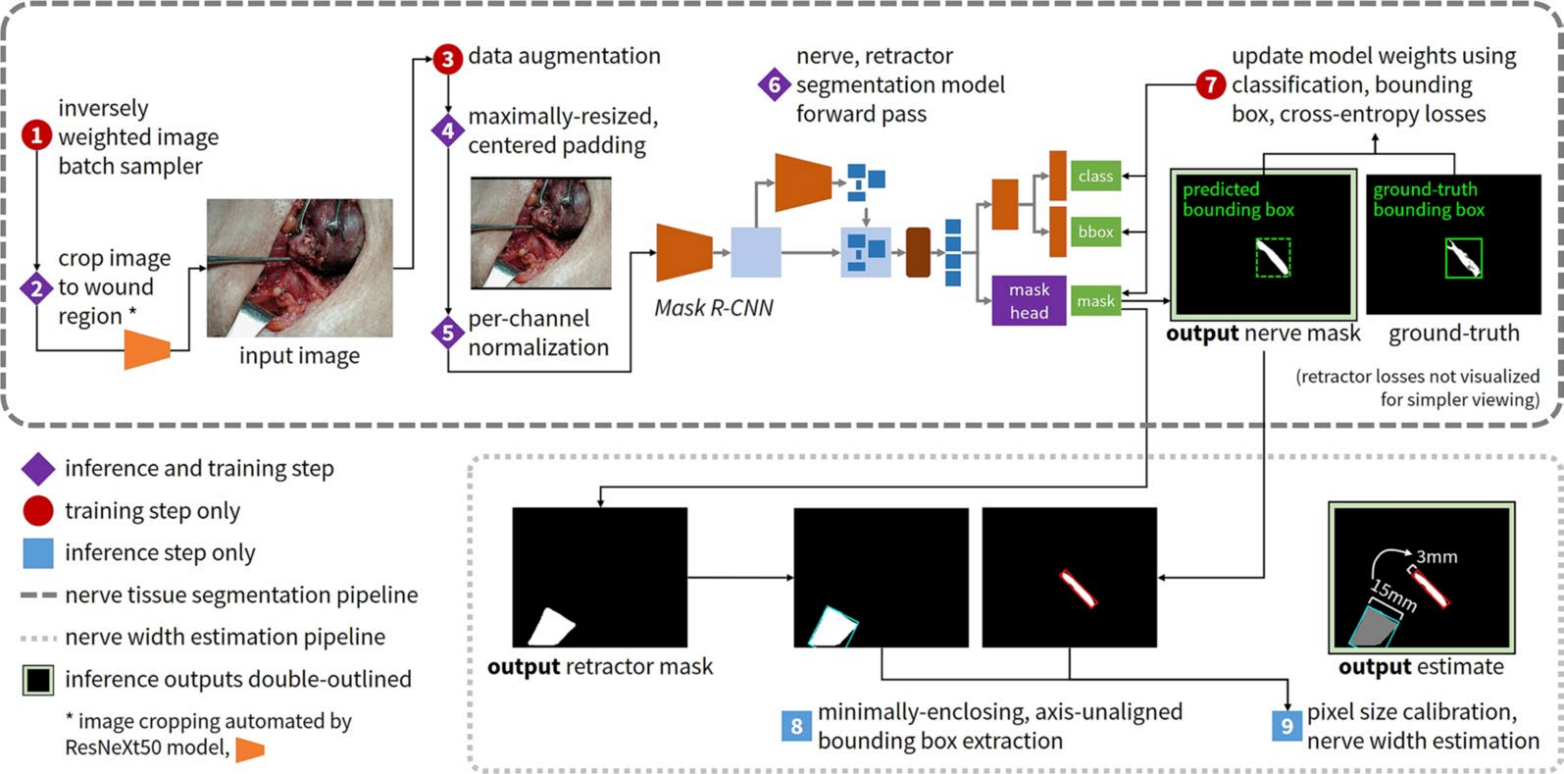
Illustration of end-to-end training and inference pipeline. Full training and inference pipeline of the proposed method for the joint nerve segmentation and measurement task. Best viewed in color. For nerve segmentation (upper half, in dark dashed box), during training, images are sampled inversely proportional to the frequency of that patient in the dataset. All images are automatically cropped using the trained bounding box predictor (where ground-truth bounding boxes for close-up images are the entire image, allowing the model to learn when and when not to crop; see Supplementary Fig. S1 for model details). Then, all images are augmented randomly via geometric and stylistic augmentation. They undergo maximally-resized, centered padding. Images are then normalized using the training dataset mean and standard deviation and run through the model to obtain the output segmentation mask. For training, the ground-truth tight, axis-aligned instance bounding box is automatically computed to minimally enclose the nerve segmentation. The output segmentation mask and ground-truth image, along with the predicted and ground-truth segmentation bounding boxes and class label (nerve), are used to compute the multi-task training loss function. The loss is back-propagated through the network to update the model weights. In inference, test images are cropped, padded, resized, and normalized as done in training, and the resulting images are passed through the model for the output segmentation. The mask prediction with the highest score is taken to be the final prediction. Next, for maximum nerve width estimation (lower half, in light dotted box), we use the same training pipeline and data preprocessing methods for the army-navy retractor segmentation model, using ground-truth retractor segmentations. The loss is the same multi-task loss function used to train the nerve segmentation model. All retractor predictions with greater than 0.5 confidence are kept. During inference, the nerve and retractor segmentations are obtained after the respective models’ forward passes. The minimally-enclosing, axis-unaligned bounding box for each segmentation is obtained. The army-navy real-world measurement of 15 mm is used along with the bounding box of the retractor to calibrate the pixel-to-millimeter ratio, which is then used to calculate the final nerve width.

**Figure 2:**
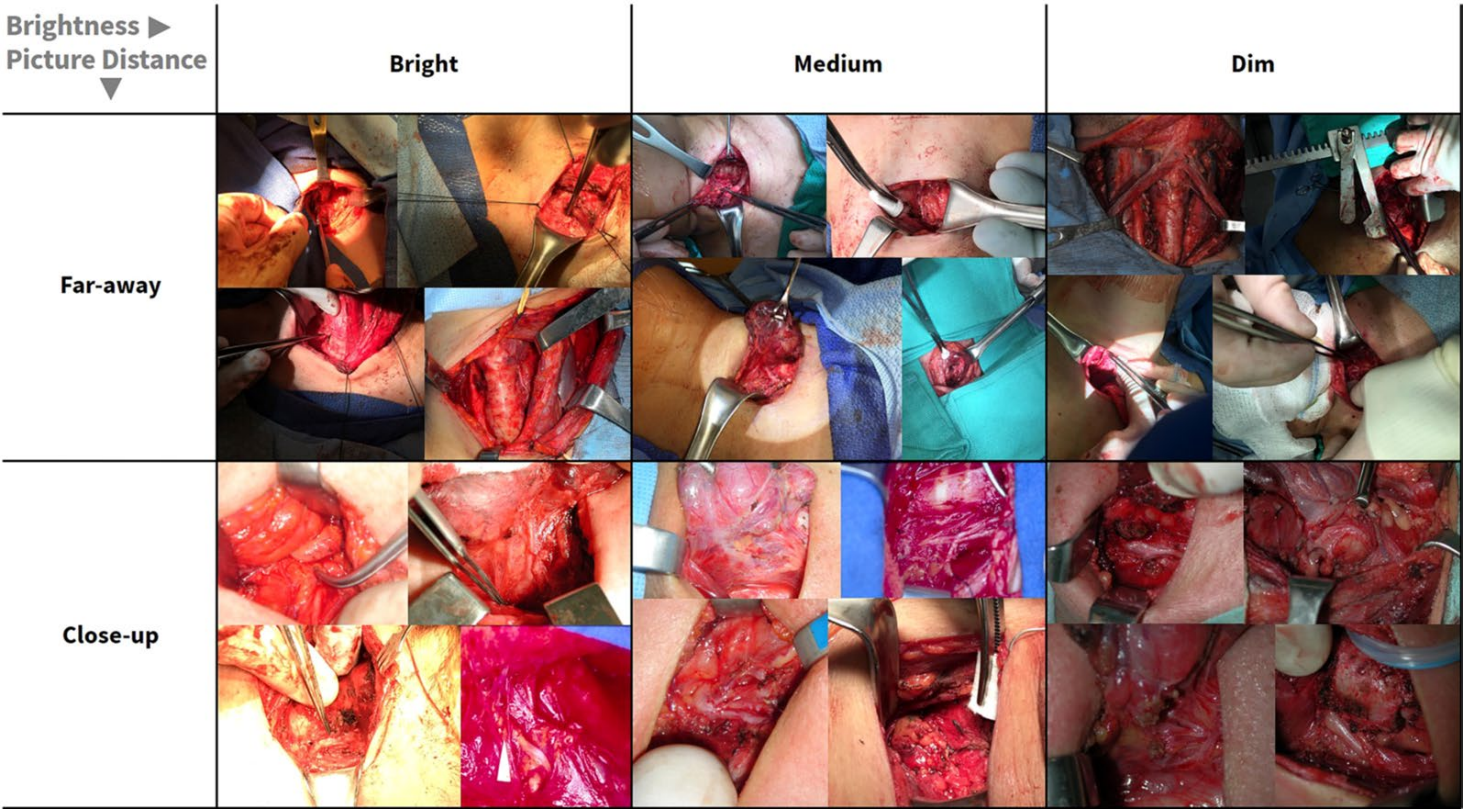
Image examples from the dataset to demonstrate diversity. Example images from the image dataset illustrate its diversity, representing diverse image conditions for lighting (bright, medium, and dim) and picture distance (far-away and close-up). Notice the high degree of variation in the images and the need for far-away perspective images to be cropped to patient anatomy to restrict the model input image to the region of interest. Note that many images are truncated to accommodate fitting inside the table cells. Best viewed in color.

We developed an end-to-end algorithm for joint segmentation and measurement of the RLN in free-form, intraoperative digital camera images (see pipeline illustrated in Fig. 1). The segmentation method leveraged weighted sampling, convolutional neural networks for cropping of the image to the wound region followed by segmentation of the nerve, and geometric and stylistic data augmentation; nerve measurement involved real-world object reference calibration (see “Methods” for details). Our dataset contains 277 color photographs representing 130 patients undergoing thyroidectomy under diverse image environmental conditions (picture distance and brightness; see dataset diversity illustrated in Fig. 2). Images are tagged with either far-away or close-up picture distance, determined by a quantitative proxy for distance (the ratio of nerve pixel area to image area; see “Methods” for details), and either bright, medium, or dim lighting. Nerve segmentations for each image were manually annotated and reviewed by head and neck endocrine surgeons.

RLN segmentations were evaluated both quantitatively and qualitatively. They were quantitatively evaluated using the dice similarity coefficient (DSC) against ground-truth surgeon annotations via k-fold cross-validation with k = 5, and under combinations of diverse image conditions, from which performance under highest-quality image conditions was also determined. The 5 cross-validation folds were randomly split by patient (see “Methods” for details). Our evaluation examines two image conditions in the dataset: picture distance and brightness. Nerve segmentations were also qualitatively examined. Nerve width estimations were evaluated via comparison against the widely-known reference range for RLN nerve width. See “Methods” for further details on the dataset, reference standard, evaluation, algorithm, and pipeline.

### Cropping model results

First, many input images (particularly those with far-away picture distances) contain irrelevant clutter in the scene and are not zoomed-in to the surgical anatomy. Thus, these images need to undergo cropping to focus on the surgical anatomy of interest before nerves can be meaningfully detected. Therefore, we use a cropping model to predict a bounding box for the wound region in the initial input image. These cropped images are then segmented by the nerve segmentation model.

We report the average precision (AP) metric for the performance of our cropping model. AP is the standard evaluation metric for object detection and ranges from 0 to 1, with good models achieving greater than 0.5 AP and stronger models even closer to 1. We calculate the AP for the cropping model using a standard object detection evaluation protocol^44^ that reports the overall AP averaged over APs at 10 different IoU thresholds, from 0.5 to 0.95 in steps of 0.05. The AP at a given IOU threshold is given by the area under the precision-recall curve of object detections at that IOU threshold. The image cropping model in our pipeline achieved a micro-averaged (all images equally weighted) AP across all 5 folds of 0.756. Subdivided by picture distance, it achieved an AP of 0.677 for the far-away image condition and 0.872 for the close-up image condition. Detailed breakdowns of the AP for each fold are in Supplementary Table S1.

### Nerve segmentation results

**Figure 3:**
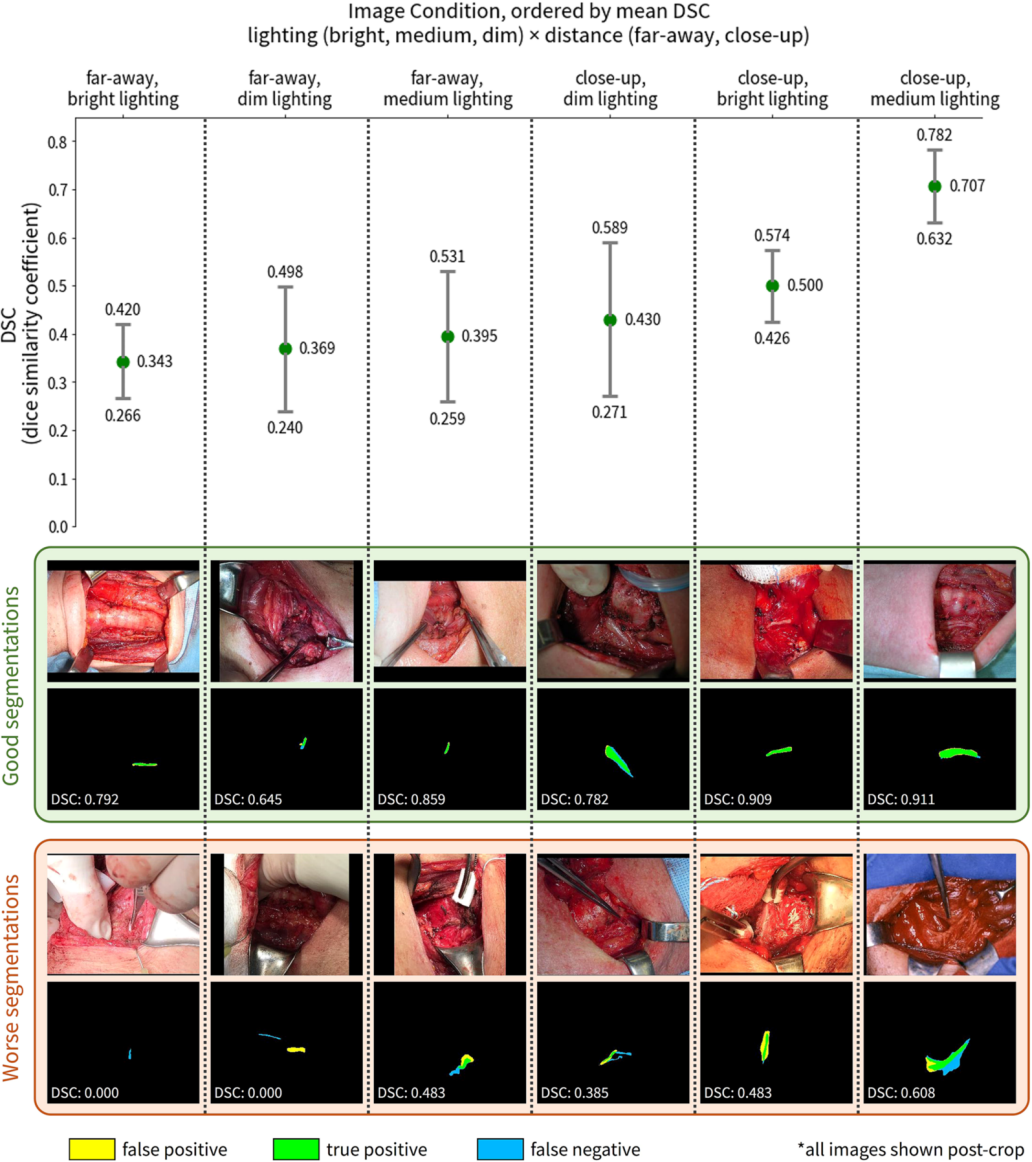
Qualitative nerve segmentation results. Qualitative nerve segmentation results under different image conditions (picture distance and brightness), with summary statistics and example images from all 5 cross-validation test folds. For each of the six image conditions labeled at the top of the figure, we show three results: a plot at the top with the mean DSC scores and 95% confidence intervals (as shown in Table 1), and in the two bottom boxes, corresponding example good (green) and worse (orange) results from each image condition to illustrate the range of model performance. For each vertical image pair in the two bottom boxes, the original image is on top and the overlaid predicted and ground-truth segmentations annotated by surgeons is on the bottom. In the overlaid segmentations, the intersection of the predicted and ground-truth segmentations (true positive) is green, false positive pixels are in yellow, and false negative pixels are in blue. Note that both far-away and close-up images and corresponding segmentations are shown post-crop with the cropping model for better segmentation visualization. In the good segmentation cases, the predicted segmentation masks track closely with the ground-truth and demonstrate successful learning of expected nerve morphology. Furthermore, notice that the algorithm performs well even in difficult scenarios such as very thin nerves (e.g. far-away, bright lighting), small nerves (far-away, medium lighting), and boundary details (close-up, dim lighting; close-up, medium lighting). The worse segmentation cases highlight challenging scenarios for the model, such as missing very small nerves (far-away, bright lighting), anatomies in better-lit portions distracting from a poorly-lit nerve (far-away, dim lighting), artifacts due to neighboring tissue texture (far-away, medium lighting), difficulty with irregular nerve shapes that deviate from more common, smooth nerve morphologies in the dataset (close-up, dim lighting; close-up, medium lighting), and light reflectance on the nerve leading to over-segmentation (close-up, bright lighting). The worse cases occur more frequently and severely in poorer distance and lighting conditions. These cases illustrate that capturing higher-quality images with respect to both picture distance and brightness is important for achieving better segmentation performance. Best viewed in color.

We next present the performance of the nerve segmentation model, which predicts segmentations from the cropped input images. The dice similarity coefficient (DSC) for segmentation is meaningful when greater than 0.5, and excellent when greater than 0.7. Under the highest-quality image conditions for picture distance and brightness (close-up distance and medium lighting) averaged across test performance of all 5 folds in the dataset, our nerve segmentation algorithm achieved a DSC of 0.707 (± 0.075, n = 40, 95% CI). DSC scores across lighting conditions ranged from 0.343 (± 0.077, n = 78, 95% CI) to 0.707 (± 0.075, n = 40, 95% CI). Confidence intervals are calculated using the standard error of the n averaged dice scores. The DSC evaluation results across each of the stratified image conditions with micro-averaged k-fold cross-validation is illustrated in Table 1. See Supplementary Table S2 for individual score breakdowns for each test fold. We also present qualitative examples of both good and worse segmentation results in Fig. 3. Overall, the good predicted segmentation masks demonstrate successful learning of expected nerve morphology, while the worse segmentation cases illustrate that the quality of image conditions strongly impacts model performance (see Fig. 3 for details). Our results show that our approach is most effective under the close-up distance and medium lighting condition, and the worse performance in other conditions illustrates the importance of high-quality image capture for optimal segmentation performance.

**Table 1:** Quantitative nerve segmentation results.

Nerve Segmentation DSC	Bright lighting 0.421 (± 0.054, n = 155)	Medium lighting 0.573 (± 0.079, n = 70)	Dim lighting 0.395 (± 0.097, n = 52)
Far-away 0.360 (± 0.058, n = 138)	0.343 (± 0.077, n = 78)	0.395 (± 0.136, n = 30)	0.369 (± 0.129, n = 30)
Close-up 0.548 (± 0.054, n = 139)	0.500 (± 0.074, n = 77)	**0.707 (± 0.075, n = 40)**	0.430 (± 0.159, n = 22)

Quantitative results of micro-averaged segmentation dice similarity coefficient (DSC) across all 5 cross-validation test folds. Evaluation examines all combinations of image conditions (picture distance and brightness) with 95% confidence intervals. Confidence intervals are calculated using the standard error of the n averaged dice scores. Best-performing condition is bolded. The algorithm had the best performance on images that were close-up to the surgical anatomy and had medium lighting.

### Nerve width estimation results

Nerve width is a useful indicator of nerve location and health, and has widely-known reference ranges in the clinical community that we use for evaluation. Our joint pipeline that performs maximum nerve width estimation illustrates the power of nerve segmentations to provide medically relevant insights when calibrated with a real-world setting. We first segmented an army-navy retractor in the surgical scene, and then used the real-world reference of its known 15 mm width, along with the predicted nerve segmentation, to calculate the nerve width (see the lower half of Fig. 1 for the nerve width estimation pipeline, and Methods for pipeline details).

**Figure 4:**
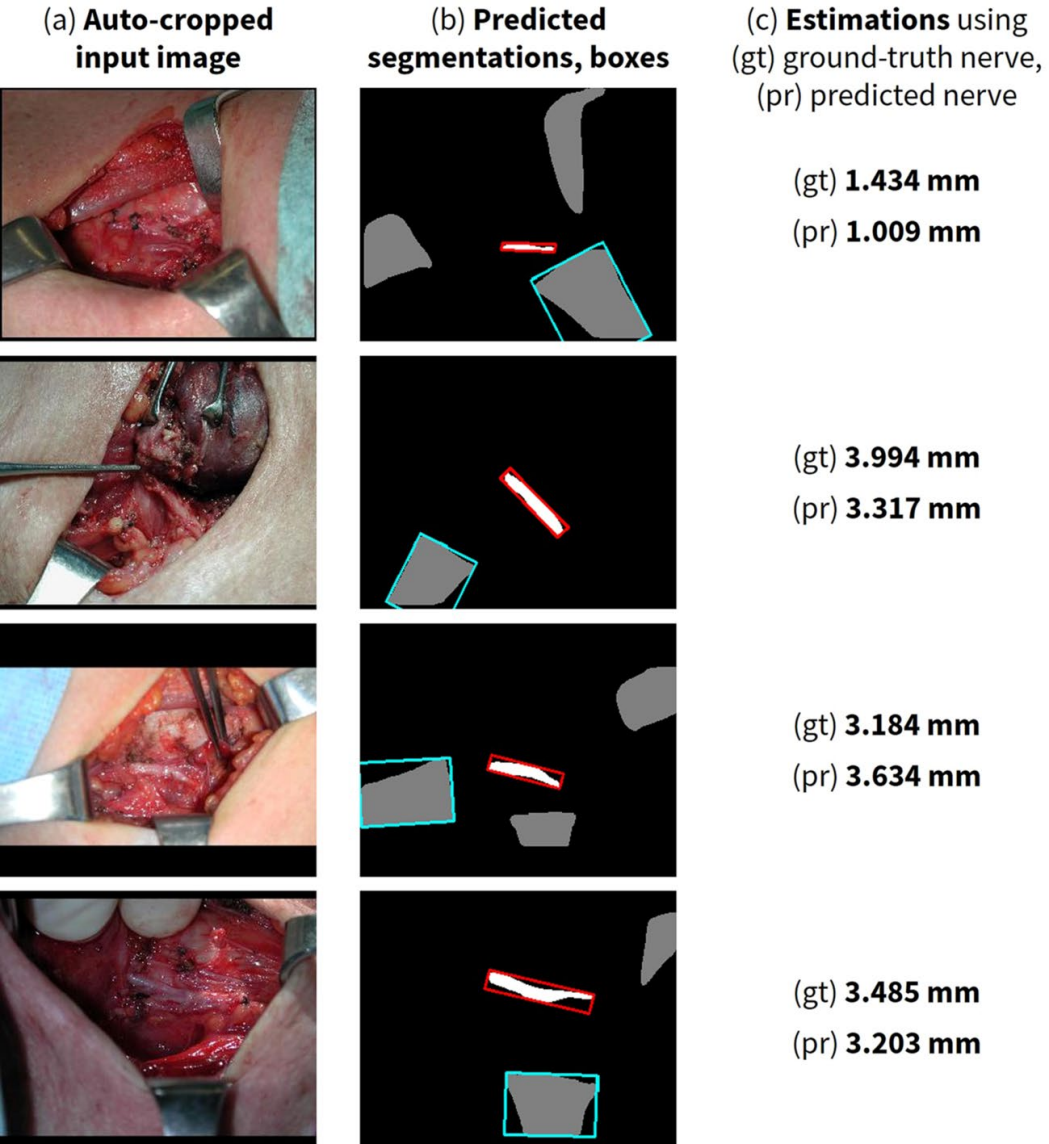
Nerve width estimation with qualitative and quantitative evaluation. Examples of nerve width estimations with (**a**) the automatically cropped original input image, (**b**) predicted segmentations and corresponding bounding boxes for nerve in white and red respectively, and for retractor in gray and blue respectively, and (**c**) estimated maximum nerve width using the reference retractor width of 15 mm and (gt) ground-truth nerve segmentations and (pr) predicted nerve segmentations (predicted retractor segmentations are used across both estimates for consistency, as some images did not have ground-truth retractor annotations). Overall, the estimations derived from both ground-truth and predicted nerve segmentations are similar. *Nota bene*: these representative images of the RLN are taken from a variety of anatomic locations within the paratracheal groove, from thoracic inlet to close-by the cricothyroid joint, and the estimates reflect this variety. Best viewed in color.

In Fig. 4, we illustrate several test images that contain army-navy retractors, along with the maximum width estimates derived from both ground-truth and predicted nerve segmentations. Since some images did not have ground-truth retractor annotations, predicted retractor segmentations are used across all estimates for consistency. Note that this method using minimally-enclosing, axis-aligned bounding boxes measures an upper bound on the nerve width. The range of measured nerve widths of 1-4 mm from intraoperative photographs falls within previously published measurements of cadaveric studies of the RLN, 1-7mm^45,46^. Moreover, using this method, the estimates closely approximate those derived from the ground-truth segmentations. Differences between ground-truth measurements and predicted estimates are less than 1 mm, i.e. beyond the detection of unaided human vision.

## Discussion

In this study, we develop a deep learning algorithm to identify the recurrent laryngeal nerve, as well as to estimate its width using free-form, digital image capture. We further analyze the impact of picture distance and lighting conditions on its performance. Through 5 fold cross-validation, the resulting deep learning segmentation algorithm achieved strong performance in the highest-quality image condition (close-up distance and medium lighting), with an average dice similarity coefficient (DSC) of 0.707 (± 0.075, n = 40, 95% CI) averaged across test performance of all 5 folds. The range of DSC scores was 0.343 (± 0.077, n = 78, 95% CI) to 0.707 (± 0.075, n = 40, 95% CI) across the whole spectrum of image conditions, which illustrates the effect of image conditions on model performance. The nerve width estimation results also show the ability of our algorithm to measure surgical anatomy using these predicted nerve segmentations. The wide range of performance across image conditions shows that proper image capture techniques play an important role in developing an effective nerve segmentation algorithm for open surgery. The strong performance in the highest-quality image condition suggests the capacity of our nerve segmentation algorithm to generalize to the complex environment of open surgery anatomy when captured appropriately by free-form digital cameras.

Many prior reports on segmentation of medical images have focused largely on structured biomedical images and radiological scans^47–51^. However, leveraging these models’ success to soft tissue segmentation in digital open surgery images is not trivial, posing unique challenges due to the surgical setting and freehand digital images. While previous works in anatomical segmentation of surgical images have mainly examined laparoscopic and robot-assisted surgery^36,38,39^, these procedures generally have more constrained surgical scenes and standardized forms of image capture. Using a more accessible but freeform imaging modality in the unconstrained environment of open surgery brings lack of image structure and anatomical structure, lack of appearance consistency, and heightened difficulty of anatomical identification.

Our algorithmic solution shows promising results despite these challenges. We achieve accurate identification of the RLN in images taken freehand by surgeons under the optimal image conditions of close-up capture distance and medium lighting, which suggests that standardizing image capture distance and lighting can be useful for achieving strong segmentation performance in intraoperative color photographs of open surgery. This result is encouraging since the RLN can be challenging to identify in these unstructured images due to its size, variable branching^52^, and complex three-dimensional relationships among multiple soft tissues. We also hope that our examination of the impact of image conditions on anatomical identification in the open surgery setting will benefit future work in this area.

While our deep learning algorithm exhibits promising performance on nerve identification and width estimation and yields important insights, there are some limitations. First, our dataset contains 277 images from 130 patients. While this collection of images taken following thyroidectomy would be, to our knowledge, the largest such dataset, this is a relatively small sample size for supervised deep learning. Despite this size limitation, the strengths of the dataset lie in its diversity in image conditions; our results demonstrate that our method that leverages weighted sampling and diverse data augmentation techniques can provide strong performance in high-quality image conditions, as well as some generalizability across the whole spectrum of various image conditions. Next, our algorithm does not always catch the precise border of the nerves, especially those that are much smaller than average, not as exposed in the image, have irregular shapes, or have nearby environmental distractors such as bright light reflectance or poor lighting (see Fig. 3 for examples and details). We observe that both picture distance and lighting conditions can create these challenging scenarios and have a noticeable impact on the algorithm’s performance. However, we show that images with the optimal conditions of close-up capture distance and medium lighting can mitigate these issues to yield better segmentations. As such, our results show that improved image capture during surgery (standardized capture distance and lighting) may help to further refine this computer vision and AI method to assist the surgeon. It may also be beneficial to use additional data augmentation methods and to incorporate medical prior knowledge to improve this method. Our nerve width estimation method can also be improved to take into account camera and object pose. Taken together, from image capture to analysis and interpretation, we show the feasibility of an end-to-end approach that surgeons could one day bring to the operating room. Finally, this study focuses on images taken after completion of thyroidectomy; we expect collecting more data during all stages of the operation will improve actionable anatomic recognition throughout the procedure.

Thus, continued progress in computer vision, deep learning, and AI are poised to develop further the concept of precision surgery. Indeed, the advent of such technology to augment a surgeon’s judgment may be arriving just in time. With recent changes in surgical training, studies have shown that residents have less time in the operating room, lower operative volumes^53^, and reduced autonomy^54^, leading to diminished ability to develop their own human visual algorithms. AI tools in the operating room may one day help to improve the learning curve outside formal training within residency. And for surgeons already in practice, computer vision and AI also have relevant applications. The link between volume and quality is well-documented in a variety of complex procedures, including thyroidectomy^7,8^. Our method demonstrates the feasibility of using computer vision and AI, perhaps one day in real-time, to identify and protect critical structures, effectively refining the outcomes of the occasional thyroid surgeon to those of an “expert” level surgeon.

Altogether, we believe this study demonstrates the feasibility of integrating computer-based algorithms into surgical vision and augmenting intraoperative decision-making. We further illustrate the important role that good image capture technique plays in the relatively unstudied realm of AI-assisted open surgery. This work heralds the promise of identifying critical anatomy in surgery, and points to the value of ﻿﻿real-time artificial intelligence in the surgical setting. These findings pave the way for implementing augmented, mixed, and extended reality in the operating room to improve surgical discrimination and patient outcomes.

## Conclusion

We present a deep learning algorithm to identify and measure the RLN as an end-to-end pipeline from free-form image capture, to automatic cropping, to segmentation, and finally to analysis. These promising results suggest the potential for deep learning to provide insight to the operating surgeon, along with the importance of image capture technique for achieving high performance. Moving forward, we envision this work as a first step toward reliable and real-time soft tissue discrimination, opening up opportunities to integrate freely moving, intelligent tools into surgery—augmenting and improving the surgical workflow while providing actionable insights.

## Methods

This study utilizes retrospectively acquired, de-identified images, as described in the Image Dataset section. Ethical approval was granted by the Institutional Review Board (The Administrative Panels on Human Subjects in Medical Research in the Research Compliance Office) of Leland Stanford Jr. University. All methods were carried out in accordance with IRB guidelines and regulations. Since these images were archival, retrospectively collected images, a waiver of consent was approved by the Institutional Review Board of Leland Stanford Jr. University under eProtocol #56217.

### Image dataset

After thyroidectomy, 277 color (RGB; red–green–blue) photographs were taken to document surgical anatomy. These digital images were obtained using a digital SLR (Nikon 3000) and smartphone (iPhone, Apple) from 2013 to 2020 at Stanford University, along with retrospectively collected images from previously published work^52^. They represent a variety of procedures, including lobectomy and total thyroidectomy, with and without central compartment and lateral neck dissection. Thus, these archival images represent a diverse array of perspectives on surgical anatomy, including the RLN and adjacent soft tissues. Images were labeled with meta-tags for image conditions of lighting and picture distance. Lighting refers specifically to the brightness of the lighting used to illuminate the target surgical anatomy, since overall image brightness may differ within the whole surgical scene. To define picture distance, we first calculate the ratio of the ground-truth nerve segmentation pixel area to the whole image area; this relative ratio is a quantitative proxy for the distance at which the picture was taken. We then divide the ratios into two buckets; namely, the lower and upper 50% of the distribution. We refer to the lower-ratio bucket as the far-away condition, and the higher-ratio bucket as the close-up condition. The range of ratios was from 0.007% to 5% of the image. Due to the high diversity of images in the dataset, the distribution of these ratios reflects the distribution we expect to see in diverse RLN images in real-world settings. In total, the final dataset represented 277 images taken from 130 patients. Notice the diversity of dataset image environmental conditions in Fig. 2.

### Reference standard

Ground-truth annotations for each surgical image were created via a two-step process: first, three head and neck endocrine surgeons used an online tool (see Supplementary Fig. S3) to carefully manually annotate each image for the RLN, as well as other aspects of the surgical scene. A senior surgeon (F.C.H.) then reviewed and confirmed each annotation. Surgeons also manually annotated many, but not all retractors in the images. Images were randomly split by patient into 5 cross-validation folds to ensure each patient’s images only appeared in one fold. Respectively, folds 1–5 contain 52 images (25 patients), 68 images (26 patients), 48 images (26 patients), 73 images (26 patients), and 36 images (27 patients). Each randomly sampled fold contains images across different lighting and picture distance levels in the image meta-tags, which yields a challenging evaluation of the model’s generalization to these different settings.

We then automatically constructed tight, axis-aligned ground-truth bounding boxes for each nerve (corresponding to nerve instances) using these ground-truth semantic nerve segmentations, since the vast majority of nerve segmentations in our dataset contain a single segmentation. To handle the semantic retractor ground-truth annotations, individual retractor connected components were first extracted from the annotations as ground-truth instances, and the corresponding instance bounding boxes were then automatically generated in the same way they were for the nerve segmentations. See Supplementary Methods for details on the ground-truth label generation process.

We also manually annotated ground-truth bounding boxes for 136 of the images to use for training the image cropping model. For the remaining images, the bounding boxes were automatically set to be the corners of the image.

### Task and evaluation metrics

The two tasks in this study were nerve segmentation, or pixel-level labeling of the nerve tissue, in each digital surgical image, as well as maximum nerve width estimation using these segmentations. We present a unified pipeline for nerve segmentation and measurement (see Fig. 1).

The primary metric for the segmentation task was the dice similarity coefficient (DSC), functionally the F1 score. It is formulated as follows with respect to the predicted and ground-truth pixel-wise segmentation labels, with TP denoting true positive, FP false positive, and FN false negative:

$$DSC=\frac{2TP}{2TP+FP+FN}^{55}.$$

This metric is the widely-accepted standard in computer vision for segmentation evaluation and is a stringent measure of how well the predicted and ground-truth segmentations overlap, balancing precision and recall. Note that since the images in our dataset generally contain one nerve, we use the DSC to capture segmentation performance. Though we use an instance segmentation model for nerve segmentation, the final output prediction is fixed to be the mask prediction with the highest score; therefore, the DSC score evaluation is on this highest-scoring mask prediction.

On the segmentation task, we also perform two sets of analysis. First, we evaluate the overall combined performance of the model on the 5 held-out cross-validation test folds. Next, we stratify the folds along the two image conditions: brightness (bright, medium, and dim) and proximity of the camera to the surgical anatomy (far-away and close-up perspectives). We rigorously evaluate the model on each of these settings to examine the algorithm’s performance under combinations of these image conditions. These results illustrate the potential of high-quality image conditions to boost segmentation performance, which we discuss. In “Results”, we also report the performance (average precision) of the cropping model used to crop images to the wound region prior to segmentation.

To demonstrate the clinical value of nerve segmentations, we perform inference of the maximum width of a given nerve tissue based on the extracted segmentation in the first task. This metric is an indicator of nerve location and health, and has widely-known reference ranges in the clinical community that we use for evaluation. We estimate nerve width by leveraging the nerve segmentation, along with the segmentation of a reference surgical tool with a standard size, the army-navy retractor.

### Segmentation algorithm

We created an end-to-end pipeline for data ingestion, processing, training, and inference. We conducted k-fold cross-validation with k = 5 on all steps of the training and inference pipeline for both the cropping model that crops input images to the wound region and the segmentation model that outputs the final nerve segmentations.

### Data annotation and ingestion

For data ingestion, surgeons used an online manual annotation tool, along with computer tablets and styluses (Wacom Co., Ltd; Saitama, Japan), to annotate fine-grained manual segmentations and cropping bounding boxes. Since images varied in both lighting and picture distance, we also labeled meta-tags for image environmental conditions to characterize this diversity. As discussed in the Image Dataset section, each image was tagged with two image conditions: picture distance (far-away and close-up) and lighting brightness (bright, medium, and dim). These 277 total images containing the RLN, along with their corresponding nerve and retractor annotations, cropping bounding boxes, and image condition meta-tags, comprised the final dataset.

### Segmentation training and inference pipeline

After constructing the dataset, we performed data processing. Since images may contain distracting clutter, they must first be cropped to the wound region before predicting nerve segmentations. We trained a cropping model to crop the initial input images using the labeled ground-truth bounding boxes. The cropping model employs a ResNeXt50-32 × 4d convolutional neural network^56^ backbone to predict the cropping bounding box for all images automatically, trained with the soft Jaccard and L1 losses (see Supplementary Fig. S1 for model architecture and Supplementary Methods for training details). Next, we pre-processed the automatically cropped images by using maximally-resized and centered zero-padding and per-channel normalization (see Supplementary Methods for details).

At training time, cropped images underwent significant data augmentation to strengthen the diversity of images presented to the model with respect to orientation, viewpoint, lighting, and other qualities (augmentation was applied prior to padding and normalization). Since preliminary experiments revealed that leveraging images exhibiting varying clutter, resolutions, and especially picture distance and brightness, was beneficial to model performance, our final data augmentation strategy addressed these characteristics. We applied both geometric augmentation (including scaling, rotations, perspective transforms, horizontal and vertical flips, and random crops and resizing) and stylistic augmentation within a plausible range (including image brightness, contrast, saturation, and hue).

Since some patients are represented in the dataset with more than one image, we leveraged a balanced sampling technique that exposes the model to images from each patient with similar frequencies (see Fig. 1, step 1). Specifically, the sampling weight of each image was assigned to be inversely proportional to the total frequency of the images for that patient in the training dataset. This exposes the model to the full depth and breadth of surgical anatomy encountered in the dataset during model training.

The segmentation model used to identify the RLN was the Mask R-CNN convolutional neural network^57^. Originally introduced for object instance segmentation, this architecture extends the Faster R-CNN^58^ object detection model with a fully convolutional mask prediction branch to localize and segment objects. This model also leverages the signal from both object bounding boxes and segmentation masks, and its region proposal approach enables the model to localize small objects effectively, which makes it well-suited for RLN segmentation. We use the same multi-task training loss defined by Mask R-CNN^57^, which has three components: the binary cross-entropy mask loss, binary cross-entropy classification loss, and smooth L1 bounding box localization loss^59^. While we also tried other model architectures, the Mask R-CNN neural network yielded the best performance. See details of the model architecture in Supplementary Fig. S2.

At inference time, a test image is cropped, padded, resized, and normalized as during training and fed through the Mask R-CNN neural network. Since Mask R-CNN generates multiple mask predictions, the mask prediction with the highest score is taken to be the final predicted mask. The final output masks are binarized with a threshold of 0.5 (pixels are deemed positive if the output logits are greater than 0.5). The training and inference pipeline is shown in the upper half of Fig. 1 (see Supplementary Methods for details). Given the smoothness and overall lack of artifacts in the segmentations predicted by the Mask R-CNN model, no post-processing is performed on the model’s output segmentation.

### Nerve width estimation algorithm

To perform nerve width estimation, we identified an army-navy retractor in the scene and took advantage of an estimate of its known size (15 mm width) along its outermost edge to serve as a real-world reference for the nerve size.

First, we trained a Mask R-CNN retractor segmentation model^57^ with ground-truth annotations collected from surgeons, using the same training algorithm and image dataset as described above. See Supplementary Methods and Supplementary Fig. S2 for details on the architecture and losses used. For a given test image, we used our trained models to obtain segmentations of both the retractor and nerve using the algorithms described above. For retractor segmentations, all predictions with scores greater than 0.5 are kept. Then, for nerve measurement, if multiple retractors or nerve segments were detected, the largest-area connected component in the segmentation was chosen for each.

Next, we extracted the minimally-enclosing, axis-unaligned bounding box of the nerve and retractor segmentations. The smaller edge of the nerve’s extracted bounding box was taken to be the width of the nerve in pixels at its widest point, or the maximum nerve width. Then, we used the known size of the army-navy to obtain a real-world size reference calibration for the pixels in the image. Finally, we estimated the nerve width by applying this calculated ratio of pixels to real-world units (millimeters) to the smaller dimension of the nerve’s bounding box. See the lower half of Fig. 1 for our end-to-end method, and Fig. 4 for example estimation results.

## Supplementary Information

Supplementary Information.

## Data Availability

The data that support the findings of this study are available from the corresponding authors (F.C.H. and S.Y.). The data will be made available upon request.

## Code availability

The code that supports the findings of this study will be made publicly available.
