# Supplementary material for: Using deep learning to identify the recurrent laryngeal nerve during thyroidectomy
Source: Sci Rep. 2021 Jul 12;11:14306. doi: 10.1038/s41598-021-93202-y (PMC8275665; doi:10.1038/s41598-021-93202-y)
Supplement: Supplementary file 1 — Supplementary Information. [file 41598_2021_93202_MOESM1_ESM.pdf]

Using Deep Learning to Identify the Recurrent Laryngeal Nerve during Thyroidectomy

Julia Gong[1]
F. Christopher Holsinger[2] *
Julia E. Noel[2]
Sohei Mitani[2,3]
Jeff Jopling[4]
Nikita Bedi[2]
Yoon Woo Koh[5]
Lisa A. Orloff[2]
Claudio R. Cernea[6]
Serena Yeung[1,7,8] *

[1] Department of Computer Science, Stanford University, Stanford, CA, USA.
[2] Division of Head and Neck Surgery, Department of Otolaryngology, Stanford University, Stanford, CA, USA.
[3] Department of Otolaryngology-Head and Neck Surgery, Ehime University Graduate School of Medicine, Shitsukawa, Toon, Ehime, Japan.
[4] Department of Surgery, Stanford University, Stanford, CA, USA.
[5] Department of Head and Neck Surgery, Yonsei University School of Medicine, Seoul, Korea.
[6] Department of Surgery, University of São Paulo Medical School, São Paulo, Brazil.
[7] Department of Biomedical Data Science, Stanford University, Stanford, CA, USA.
[8] Clinical Excellence Research Center, Stanford University School of Medicine, Stanford, CA, USA.

*** Co-Corresponding Authors:**

F. Christopher Holsinger[2]
Division of Head and Neck Surgery, Stanford University
875 Blake Wilbur Drive, Stanford, CA 94305, USA
Email: holsinger@stanford.edu

Serena Yeung[1,7,8]
Departments of Biomedical Data Science and Computer Science, Stanford University
350 Jane Stanford Way, Stanford, CA 94305, USA
Email: syyeung@stanford.edu

SUPPLEMENTARY MATERIAL

Supplementary Methods. Training details.

Data pre-processing.

First, vertical images (images with larger height than width) were rotated 90 degrees before being processed by the cropping model. Then, they underwent maximally-resized and centered zero-padding (resizing the image and padding such that only one dimension of the image requires padding, maximizing the coverage of meaningful pixels in the resulting image). Padded images were then resized to the same standard 320 by 256 pixel dimensions. Finally, the images were pre-processed via image normalization using the per-channel (red, green, blue) means and standard deviations across images in the training dataset. All images were then automatically cropped using the cropping model. The regressed bounding box corners were used to crop the image. For segmentation, the same pre-processing steps (except for vertical image rotation) were applied to the cropped images before being fed to the segmentation model.

Mask R-CNN ground-truth label generation.

To train the nerve segmentation model, tight, axis-aligned ground-truth instance bounding boxes were automatically generated by computing the minimum and maximum x and y values in the nerve segmentation mask. For the retractor segmentation model, connected component contours for individual connected components were first extracted from the semantic segmentations to create ground-truth instance segmentations (small connected component artifacts less than 4200 pixels in area in the original, unresized images were removed). Then, tight ground-truth bounding boxes for each retractor instance were automatically computed in the same way as done for the nerve segmentations. The retractor segmentation model was only trained on images that contained retractor segmentations (246 of the 277 images).

Objective functions, hyperparameters, experiment details.

The Mask R-CNN losses for nerve segmentation are stated in the main text; the feature pyramid backbone was pretrained on ImageNet. The optimal hyperparameters were a batch size of 8 and a starting learning rate of 0.0001 (1e-04), with a schedule of halving the learning rate after a plateau of 50 epochs for 100 epochs of training and early-stopping on the epoch with the best performance on the validation set. For all other hyperparameters, we used the default values in the PyTorch library implementation of Mask R-CNN (100 box detections per image, 0.7 for the region proposal network NMS threshold, etc.; please see the official code repository for the full implementation). For the retractor segmentation model, the Mask R-CNN losses, backbone, pretraining, hyperparameters, and learning rate schedule were the same.

The cropping model loss function is formulated as an equally weighted (simple sum) combination of the soft Jaccard loss on the predicted bounding box area and the L1 loss on the four coordinates of the bounding box, (minimum x, minimum y, maximum x, maximum y), with optimal hyperparameters being a learning rate of 0.0001 (1e-04) and a batch size of 8, and a schedule of halving the learning rate after a plateau of 50 epochs for 600 epochs of training (also early-stopping on the epoch with best validation performance). The ResNeXt50 backbone is pretrained on ImageNet.

All experiments were conducted using a single Titan RTX GPU with 24GB RAM.

Cropping Model AP @ IOU=[0.5:0.95:0.05]	**Fold 1**<br>0.678 (n=52)	**Fold 2**<br>0.774 (n=68)	**Fold 3**<br>0.796 (n=48)	**Fold 4**<br>0.725 (n=73)	**Fold 5**<br>0.889 (n=36)
**Far-away**<br>0.677 (n=138)	0.616 (n=31)	0.672 (n=31)	0.732 (n=26)	0.692 (n=43)	0.741 (n=7)
**Close-up**<br>0.872 (n=139)	0.807 (n=21)	0.896 (n=37)	0.897 (n=22)	0.813 (n=30)	0.942 (n=29)
**Overall** (n=277)	**0.756**				

Supplementary Table S1. Quantitative evaluation of cropping model. Quantitative evaluation of the image cropping model across the picture distance image environmental condition, both overall and for each of 5 cross-validation test folds. We calculate average precision (AP) of the cropping model using the standard evaluation protocol of reporting the overall AP averaged over APs at 10 different IOU thresholds from 0.5 to 0.95 in steps of 0.05. All averages are micro-averages that equally weight all images. The overall cropping model AP is bolded.

DSC	**Fold** average DSC	**Bright lighting**<br>0.421 (±0.054, n=155)	**Medium lighting**<br>0.573 (±0.079, n=70)	**Dim lighting**<br>0.395 (±0.097, n=52)
	**1** 0.341 (±0.094, n=52)	0.215 (±0.109, n=30)	0.578 (±0.176, n=16)	0.333 (±0.277, n=6)
	**2** 0.484 (±0.082, n=68)	0.474 (±0.098, n=47)	0.552 (±0.215, n=14)	0.417 (±0.299, n=7)
	**3** 0.343 (±0.101, n=48)	0.394 (±0.122, n=31)	0.442 (±0.396, n=7)	0.117 (±0.188, n=10)
	**4** 0.499 (±0.080, n=73)	0.532 (±0.116, n=35)	0.532 (±0.160, n=21)	0.390 (±0.178, n=17)
	**5** 0.622 (±0.098, n=36)	0.473 (±0.221, n=12)	0.741 (±0.054, n=12)	0.652 (±0.201, n=12)
**Fold**	**Far-away**<br>0.360 (±0.058, n=138)	**Far-away and bright**<br>0.343 (±0.077, n=78)	**Far-away and medium**<br>0.395 (±0.136, n=30)	**Far-away and dim**<br>0.369 (±0.129, n=30)
**1**	0.263 (±0.116, n=31)	0.100 (±0.119, n=18)	0.544 (±0.246, n=8)	0.399 (*, n=5)
**2**	0.371 (±0.118, n=31)	0.399 (±0.131, n=24)	0.000 (*, n=4)	0.644 (*, n=3)
**3**	0.234 (±0.135, n=26)	0.297 (±0.185, n=15)	0.324 (*, n=5)	0.000 (±0.000, n=6)
**4**	0.457 (±0.109, n=43)	0.547 (±0.155, n=20)	0.341 (±0.295, n=9)	0.403 (±0.211, n=14)
**5**	0.618 (±0.262, n=7)	0.000 (*, n=1)	0.704 (*, n=4)	0.756 (*, n=2)
**Fold**	**Close-up**<br>0.548 (±0.054, n=139)	**Close-up and bright**<br>0.500 (±0.074, n=77)	**Close-up and medium**<br>0.707 (±0.075, n=40)	**Close-up and dim**<br>0.430 (±0.159, n=22)
**1**	0.455 (±0.157, n=21)	0.389 (±0.181, n=12)	0.612 (±0.319, n=8)	0.000 (*, n=1)
**2**	0.580 (±0.109, n=37)	0.554 (±0.150, n=23)	0.773 (±0.074, n=10)	0.246 (*, n=4)
**3**	0.473 (±0.144, n=22)	0.485 (±0.167, n=16)	0.737 (*, n=2)	0.292 (*, n=4)

**4**	0.558 (±0.121, n=30)	0.511 (±0.197, n=15)	0.674 (±0.164, n=12)	0.333 (*, n=3)
**5**	0.623 (±0.113, n=29)	0.516 (±0.221, n=11)	0.760 (±0.079, n=8)	0.631 (±0.245, n=10)

Supplementary Table S2. Quantitative nerve segmentation results per fold. Quantitative results of segmentation dice similarity coefficient (DSC) on each of 5 cross-validation test folds. Evaluation examines all combinations of image environmental conditions (picture distance and brightness) with 95% confidence intervals (*some image conditions within folds are small with 5 or fewer samples and have less meaningful confidence intervals, which are thus omitted). Confidence intervals are calculated using the standard error of the n averaged dice scores.

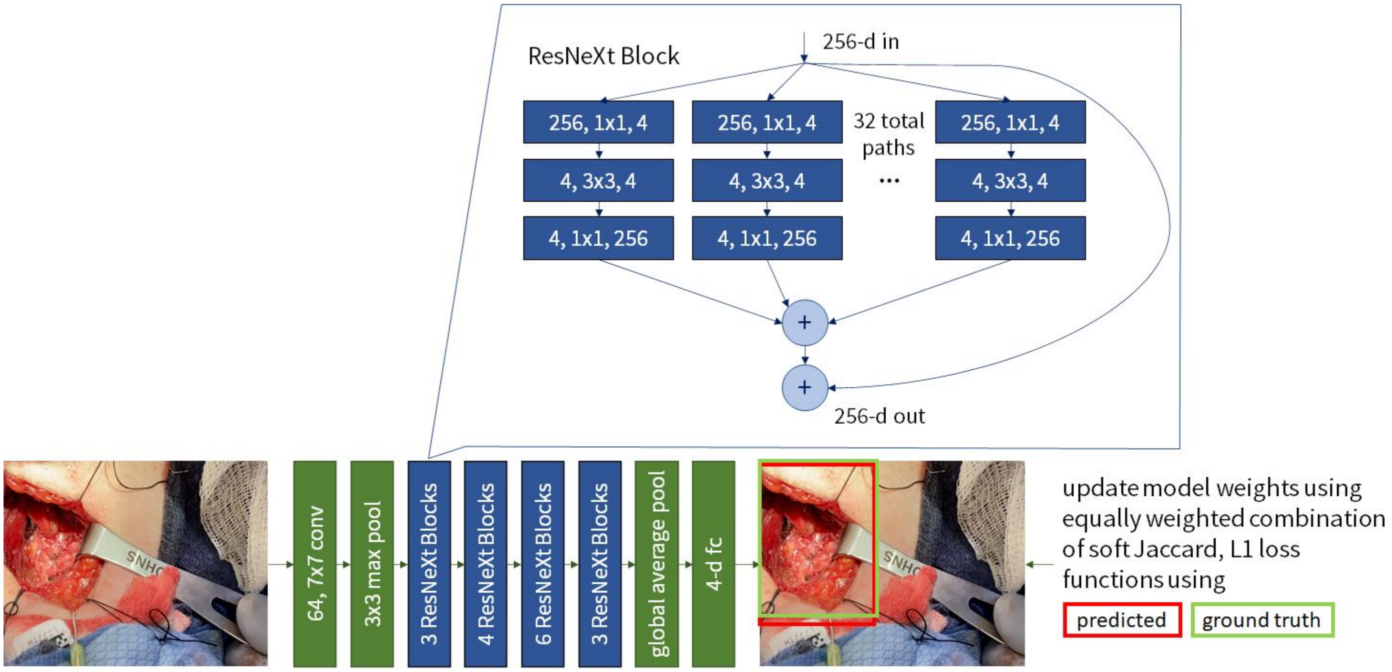

Supplementary Figure S1. Detailed cropping model architecture and training procedure. Here, we specify the detailed architecture of the ResNeXt50-32x4d model used for regressing the four values of the cropping bounding box (min x, min y, max x, max y). The network is a convolutional neural network with ResNeXt Blocks in the backbone with skip-connection paths from different feature maps of previous layers. The image is pre-processed as described in Supplementary Methods before being fed into the network. The model is trained using an equally weighted combination of the soft Jaccard and L1 losses. Best viewed in color.

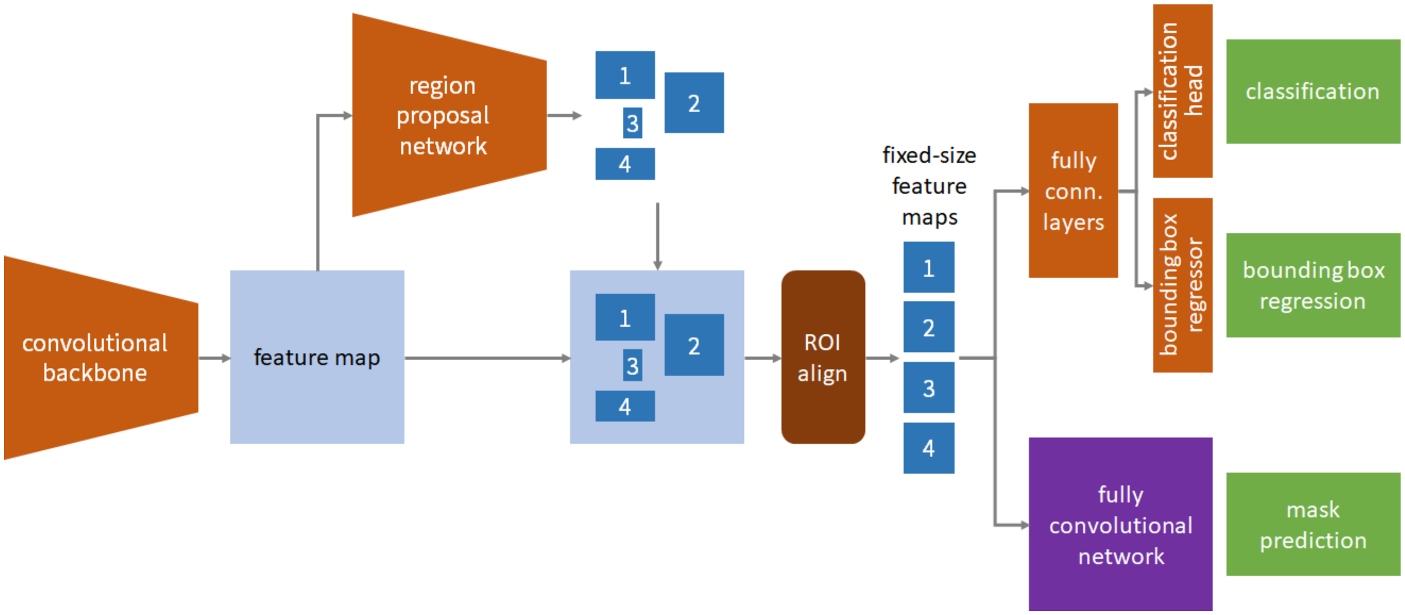

Supplementary Figure S2. Detailed segmentation model architecture. Here, we detail the architecture of the standard Mask R-CNN model with specific module information. The input images in this work are 320-by-256 pixel RGB images with 3 channels, which are passed through a convolutional feature pyramid backbone; the resulting feature map is put through another convolutional network to extract region proposals. These region proposals are processed by the region of interest (ROI) alignment layer, which produces fixed-size feature maps for each filtered object proposal. These feature maps are fed to two branches; one with fully connected layers for classification and bounding box regression, and another with fully convolutional layers for mask prediction. Best viewed in color.

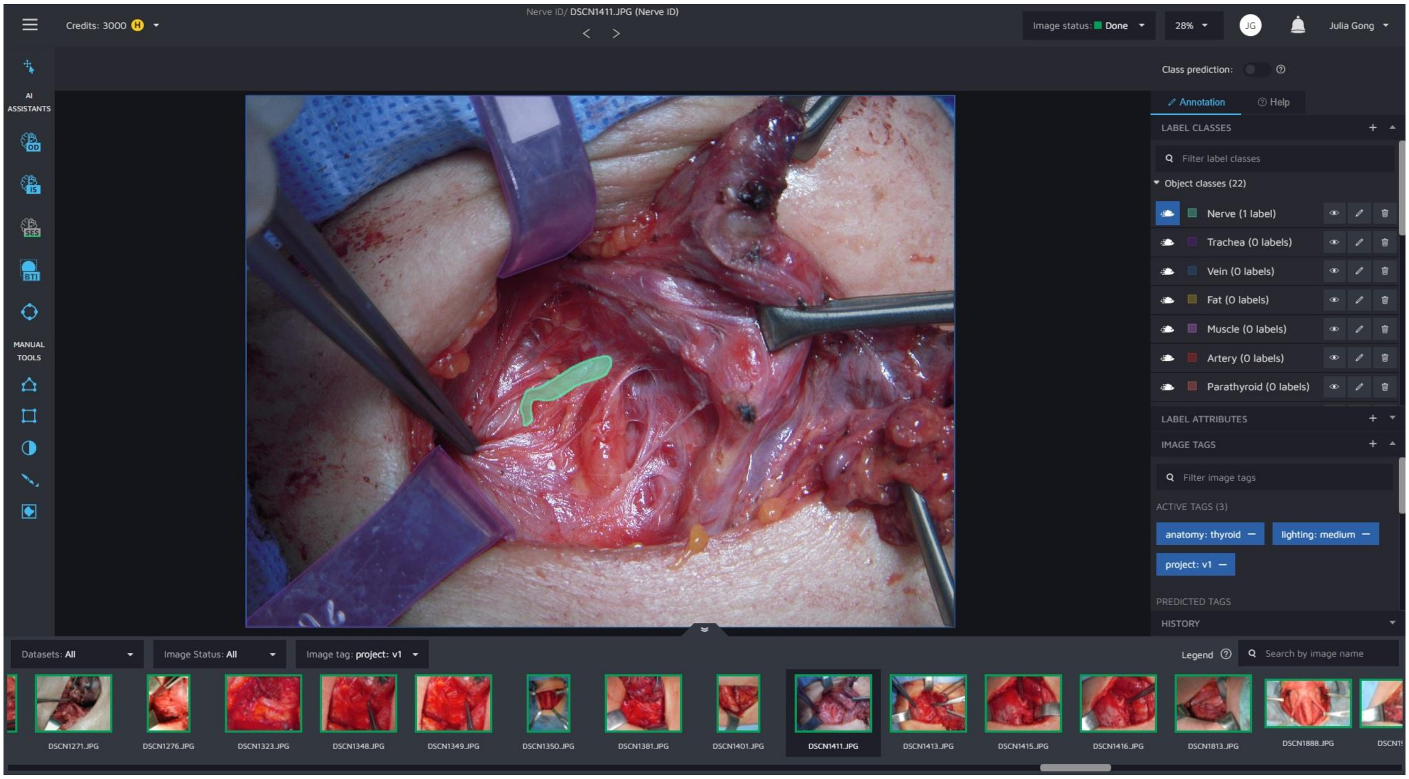

Supplementary Figure S3. Data annotation tool. A screenshot of the workflow of Hasty.ai, the online platform used for obtaining precise, manually annotated ground-truth nerve tissue segmentations from the surgeons in this study. Shown in the screenshot are the image currently being annotated in the center, with the nerve segmentation shown in green; retractors shown in purple; the panel of available images on the bottom; the annotation class selected on the upper right-hand panel, along with other attributes of interest, such as image meta-tags on the lower right-hand panel; and available annotation tools on the left-hand panel. Best viewed in color.
